# Prediction of the functional class of metal-binding proteins from sequence derived physicochemical properties by support vector machine approach

**DOI:** 10.1186/1471-2105-7-S5-S13

**Published:** 2006-12-18

**Authors:** HH Lin, LY Han, HL Zhang, CJ Zheng, B Xie, ZW Cao, YZ Chen

**Affiliations:** 1 Bioinformatics and Drug Design Group, Department of Pharmacy and Department of Computational Science, National University of Singapore, Blk SOC1, Level 7, 3 Science Drive 2, Singapore 117543; 2 Shanghai Center for Bioinformatics Technology, 100, Qinzhou Road, Shanghai 200235 P.R. China

## Abstract

Metal-binding proteins play important roles in structural stability, signaling, regulation, transport, immune response, metabolism control, and metal homeostasis. Because of their functional and sequence diversity, it is desirable to explore additional methods for predicting metal-binding proteins irrespective of sequence similarity. This work explores support vector machines (SVM) as such a method. SVM prediction systems were developed by using 53,333 metal-binding and 147,347 non-metal-binding proteins, and evaluated by an independent set of 31,448 metal-binding and 79,051 non-metal-binding proteins. The computed prediction accuracy is 86.3%, 81.6%, 83.5%, 94.0%, 81.2%, 85.4%, 77.6%, 90.4%, 90.9%, 74.9% and 78.1% for calcium-binding, cobalt-binding, copper-binding, iron-binding, magnesium-binding, manganese-binding, nickel-binding, potassium-binding, sodium-binding, zinc-binding, and all metal-binding proteins respectively. The accuracy for the non-member proteins of each class is 88.2%, 99.9%, 98.1%, 91.4%, 87.9%, 94.5%, 99.2%, 99.9%, 99.9%, 98.0%, and 88.0% respectively. Comparable accuracies were obtained by using a different SVM kernel function. Our method predicts 67% of the 87 metal-binding proteins non-homologous to any protein in the Swissprot database and 85.3% of the 333 proteins of known metal-binding domains as metal-binding. These suggest the usefulness of SVM for facilitating the prediction of metal-binding proteins. Our software can be accessed at the SVMProt server .

## Background

Metal-binding proteins play important roles in structural stability and complex formation [1-5], gene expression regulation and alteration [1,6-9], DNA processing [10], signaling processes and cellular events [11], transport [8,12,13], metabolism control [1,4,12,14], metal homeostasis [15,16], antibody recognition [17], and other events such as cellular respiration, muscle movement, and antioxidant defense [18]. Approximately 1/3 of structurally-determined proteins are metal-bound[19], and large percentages of metals present in human body are bound to proteins [4,20]. Identification of metal-binding proteins and knowledge of metal-protein interactions is important for elucidating the function and functional mechanism of proteins and biological processes.

Metal-binding proteins have been identified by such experimental approaches as absorbance spectroscopy[21], gel electrophoresis [22], metal-affinity columns and shift assay [23], chromatography [16], mass spectroscopy [22], NMR [6], and combined spectroscopic studies [24]. However, some of these methods generally require a purified or semi-purified target of interest, do not facilitate identification of unknown targets form complex protein mixtures, or require multi-step processes and very specialized equipment, which limit their application ranges [23]. Therefore, there is a need to explore other methods including computational approaches for facilitating the identification of metal-biding proteins to complement these experimental methods.

Several computational methods have been explored for identifying and characterizing metal-binding proteins. In many metalloproteins, the metal ions tightly bind to the proteins and their metal-bound structures could be accurately determined by x-ray crystallography [3,5,10,17]. Thus structural information has been used for predicting metal-binding sites based on the detection of principal liganding residues and metal-ligand complex architectures [25,26], the use of common local structural parameters [25], combination of sequence and structural profiles [27], analysis of bond strength contributions [28], and the computation of force fields [29,30]. But for those proteins with loosely or temporarily bound metals, such as enzymes that use metal ions as cofactors, the specific metal binding sites are often poorly characterized or unknown [6]. Therefore, sequence-based computational methods appear to be useful for these types of proteins and those without 3D structures. Apart from sequence similarity methods, the recently explored sequence-based methods include metal-binding sites sequence motifs [31,32], multiple sequence alignments against known metal-binding proteins[26], and neural networks of sequence segments of amino acids of higher metal-binding propensity[33]. Moreover, combinatorial use of multiple structural, sequence alignments and annotation methods has been found to be highly useful for improving prediction accuracy of metal-binding proteins[26].

Because of the sequence, structural and functional diversity of metal-binding proteins [1-5,8,9,11-15,17], it is desirable to explore additional methods that predict metal-binding proteins directly from sequence or sequence-derived properties. This work explored a statistical learning method, support vector machines (SVM), as such an approach. SVM has been successfully used for predicting the functional classes of molecule-binding proteins such as RNA-binding proteins [34,35], DNA-binding proteins [35], lipid-binding proteins [36], and transporters [37] from sequence-derived structural and physicochemical properties and irrespective of sequence similarity. Metal-binding proteins involve a substantially more diverse spectrum of proteins than most of the other classes of proteins. For instance, the zinc-binding proteins of 16,072 sequence entries belong to 765 Pfam [38] domain families, while the EC2.7 enzymes and RNA-binding proteins of similar number of sequence entries (14,171 and 14,208) belong to 548 and 378 Pfam families respectively. The diverse spectrum of proteins poses a more critical test for constructing a SVM prediction system.

Metal-binding proteins are diverse in sequence, structure, and function[1-5,8,9,11-15,17]. Nonetheless, metal cations generally bind to centers of high hydrophilicity and reduce the enthalpy of a system upon binding [30,39], and metal ions bind to a shell of polar hydrophilic residues surrounded by a shell of non-polar residues [25]. The binding sites of some metal-ligand complexes have specific structural architectures [25]. To some extent, these metal binding features are similar to those of other molecule-binding features of proteins such as RNA-binding proteins, DNA-binding proteins and transporters that are also diverse in sequence, structure and function whose binding capability are mediated by certain structural and physiochemical characteristics [36,40,41]. Therefore, it is expected that SVM is also applicable to the prediction of metal-binding proteins.

In this paper, we developed SVM prediction systems for 10 metal-binding classes and for all metal-binding proteins. These classes are calcium-binding, cobalt-binding, copper-binding, iron-binding, magnesium-binding, manganese-binding, nickel-binding, potassium-binding, sodium-binding and zinc-binding. In addition to the estimate of the prediction accuracy by using an independent set of proteins, the performance of our developed SVM prediction systems was further evaluated by four additional tests to determine the usefulness of SVM for predicting novel metal-binding proteins and the applicability of other kernel functions. One is the evaluation of the prediction accuracies when homologous proteins are considered as one. The second is the prediction of metal-binding proteins non-homologous to any protein in Swissprot database[42]. The third is to study whether the known metal-binding domains can be predicted as metal-binding by our SVM systems. The fourth is to study the performance of SVM with a different kernel function.

## Results and discussion

### Overall prediction accuracy

The statistics of the datasets and prediction results of specific metal-binding classes and all metal-binding proteins are given in Table 1. In this Table, *TP, FN, TN FP, SE*, and *SP *stand for true positive (correctly predicted metal-binding proteins of specific class), false negative (specific class of metal-binding proteins incorrectly predicted as non-class-members), true negative (correctly predicted non-class-members), false positive (non-class-members incorrectly predicted as specific class of metal-binding proteins), the predicted sensitivity (accuracy for members in each metal-binding class), and the predicted specificity (accuracy for non-members of each metal-binding class). The SE for the class of calcium-binding, cobalt-binding, copper-binding, iron-binding, magnesium-binding, manganese-binding, nickel-binding, potassium-binding, sodium-binding, zinc-binding, and all metal-binding proteins is 86.3%, 81.6%, 83.5%, 94.0%, 81.2%, 85.4%, 77.6%, 90.4%, 90.9%, 74.9% and 78.1% respectively. The corresponding SP is 88.2%, 99.9%, 98.1%, 91.4%, 87.9%, 94.5%, 99.2%, 99.9%, 99.9%, 98.0%, and 88.0% respectively.

A direct comparison with results from previous metal-binding protein prediction studies may not be most appropriate because of the differences in the protein classes predicted, datasets, protein descriptors, prediction methods and parameters. Nonetheless, a tentative comparison may provide some crude estimate regarding the level of accuracy of our method with respect to those achieved by other studies of metal-binding proteins. The reported SEs of other studies are in the range of 87%~93% for calcium-binding proteins [28] and 90~97% for all metal-binding proteins [25,27,29,33]. Thus the corresponding SEs of 93.8% and 78.9% of our SVM prediction systems are comparable to those of other studies despite of the use of a significantly higher number, and thus more diverse range, of proteins in our studies.

The prediction accuracy of the non-members of each metal-binding class appears to be better than that of the members. The higher prediction accuracy for non-members likely results from the availability of more diverse set of non-members than that of members, which enables SVM to perform a better statistical learning for recognition of non-members. Based on the statistics provided on the webpage of Pfam database [38], there are over 8,000 families of proteins, from which one can generate a diverse set of non-members for each metal-binding class. Because of the differences in the number of members and that of non-members in each class, there is an imbalance between each dataset. SVM based on an imbalanced datasets tends to produce feature vectors that push the hyperplane towards the side with smaller number of data [43], which can lead to a reduced accuracy for the set either with a smaller number of samples or of less diversity. This might partly explain why the prediction accuracy for members is generally lower than that for non-members. It is however inappropriate to simply reduce the size of non-members to artificially match that of members, since this compromises the diversity needed to fully represent all non-members. Computational methods for re-adjusting biased shift of hyperplane are being explored [44]. Application of these methods may help improving SVM prediction accuracy in this and other cases involving unbalanced data.

### Prediction of novel metal-binding proteins

One particular application of SVM is the prediction of novel metal-binding proteins that are non-homologous to other proteins [45]. To test this capability, Swiss-Prot database [42] was searched for metal-binding proteins having no single homologous protein in the database based on PSI-BLAST [46] results. A similarity E-value threshold of 0.1 was used for homolog search to ensure maximum exclusion of proteins that have a homolog. Those proteins found in the SVM training sets are then removed. As shown in Table 2, a total of 87 proteins are found from this process, 58 or 66.7% of these proteins were correctly predicted as metal-binding by our SVM classification systems respectively. Therefore, our SVM classification systems appear to show reasonably good capability for predicting novel metal-binding proteins based on the set of proteins tested. 9 of the 29 incorrectly predicted novel metal-binding proteins are nucleic acid binders. These include endonucleases Cfr10I, CviJI, EcoRV, PvuII and BslI, transcription activators chrR and rep2, meiosis-specific protein HOP1, protein suppressor of variegation 3–7. One possible reason for the misclassification of these nucleic acid binding proteins is that spatial conservation rather than sequence conservation plays the dominant role in the formation of active site where metal ions are clustered [47], which is more difficult to predict than the classes of proteins with more apparent sequence signatures.

### Prediction of metal-binding proteins with specific structural characteristics

A number of metal-binding proteins contain metal-binding domains [31,48,49] or motifs [31,32]. Several families of such metal-binding proteins have been documented, and examples of these families are zinc finger family[50], EF hand family[51], and Fer4 family[52]. These families have distinguished structural features responsible for metal-binding. Thus the performance of SVM prediction of metal-binding proteins can be evaluated by examining whether or not proteins containing one of these domains or motifs can be correctly classified as metal-binding.

A search of protein family and sequence databases showed that there are 2462, 780, and 534 metal-binding protein sequences known to contain zinc finger, EF hand, and Fer4 domain respectively. The majority of these sequences are included in the training and testing set of all metal-binding proteins. In the corresponding independent evaluation set, there are 890, 215, and 192 sequences containing zinc finger, EF hand, and Fer4 domain respectively. Most of these protein sequences were correctly classified as metal-binding by SVM. There are only 17, 8, and 6 misclassified sequences in the zinc finger, EF hand, and Fer4 domain families respectively. Thus our results showed the capability of our SVM prediction systems for recognizing these metal-binding proteins.

### Prediction performance for metal-binding domains

Some metal-binding proteins are known to contain multiple domains that include a metal-binding domain plus one or more domains characterized by DNA binding, protein-protein interaction and other motifs [53-56]. Our SVM prediction systems were trained by using physicochemical properties derived from the entire protein sequence. There is a need to evaluate how the inclusion of all the other "extra" domains may affect the prediction performance of our SVM systems. For such a purpose, our SVM systems were tested to determine to which extent they can predict known metal-binding domains as metal-binding without having to include representatives of these domains in our training sets. Metal-binding domains are searched from the Pfam database [38] by using key word of ten biological common metals- calcium, cobalt, copper, magnesium, manganese, nickel, potassium, sodium and zinc, followed by manual evaluation of the hits to select those with such annotations as experimental proved metal contained structure, metal chelating site, metal-based functional site, as well as metal channel, transporter, and carrier. A total of 333 distinct metal-binding domains are selected from this process, which include 127 domains in multi-domain metal-binding proteins. It is found that 85.3% and 81.1% of these are predicted as metal-binding. Moreover, 82.5% of the 1368 multi-domain metal-binding proteins in our independent set is correctly predicted. Hence, the inclusion of "extra" domains appears to have a limited effect on the performance of our developed SVM systems, which show certain level of capability for predicting metal-binding domains as well as metal-binding proteins.

### SVM prediction performance by using a different kernel function

Apart from the Gaussian kernel function of sequence-derived physicochemical properties used in this work, several other kernel functions have been developed and applied for SVM analysis of proteins and DNAs [57-59]. It is of interest to test the usefulness of some of these kernel functions for predicting metal-binding proteins. The string-kernel function has been extensively used and it has shown promising potential for protein and DNA studies [57,58]. This kernel function is constructed by comparison of sequences of classes of proteins or DNAs and the assignment of individual weights to amino acids or nucleotides to describe physicochemical or other characteristics of the proteins and DNAs. In this work, this kernel function is used to develop three SVM systems for predicting the class of nickel-binding proteins, potassium-binding proteins, and sodium-binding proteins. Spectrum kernel with mismatches[60] is used to generate the string-kernel for each protein. Testing results by using the independent set of proteins for each class show that the SE is 75.1%, 89.5% and 88.7%, and the SP is 99.0%, 98.7% and 97.8% for each of these classes respectively. Thus comparable prediction performance can be achieved by using string-kernel SVM, which suggests the usefulness of this and other kernel functions for SVM prediction of metal-binding proteins.

### Comparison of SVM prediction performance with those of other methods

To compare the prediction performance of SVM with those of other methods, our SVM classification system for predicting zinc-binding proteins was used to scan the human genome, and the predicted zinc-binding proteins were compared with those predicted by one or combinations of three other methods[26]. These methods have been used for searching potential zinc-binding proteins in human genome by means of (i) zinc-binding pattern identification via structural comparison with all available X-ray structural data, (ii) multiple sequence alignment based on libraries of zinc-binding domains, and (iii) analysis of sequence annotations[26]. SVM predicted a total of 4,518 zinc-binding proteins compared to that of 3,207 by at least one of the other three methods, 2,770 of which are mutually predicted. The percentages of mutually predicted proteins are significantly higher for those proteins predicted by using combinations of the other three methods. The numbers of proteins predicted by at least two and three of the other methods are 2,430 and 1,684 respectively, 2,256 and 1,615 of which are also predicted by SVM. Therefore, SVM is capable of predicting most of the zinc-binding proteins predicted by the combinations of the other three methods. SVM appears to predict a higher number of zinc-binding proteins than each of the other three methods. Apart from the expected prediction error, the reported problems of the other three methods associated with structurally uncharacterized, non-conserved, unclearly annotated zinc-binding proteins[26] may also contribute to the discrepancy between SVM and the other methods. For example, two SVM predicted proteins that are not predicted by the other three methods, forkhead box protein P1 and TRAF-interacting protein, are annotated as zinc-binding in GO and described to contain ZINC_FINGER_C2H2_1 in PROSITE.

### Contribution of feature properties to the classification of metal-binding proteins

In this work, a total of nine feature properties were used to describe physicochemical characteristics of each protein, which have been routinely used for the prediction of other molecular-binding proteins [61]. It has been reported that, not all feature vectors contribute equally to the classification of proteins, some have been found to play relatively more prominent role than others in specific aspects of proteins [62]. It is therefore of interest to examine which feature properties play more prominent role in classification of metal-binding proteins.

In an earlier study, contribution of individual feature property to protein classification is investigated by separately conducting classification using each feature property [36,40,41]. The same method was employed here. An analysis on the classification of the group of all metal-binding proteins seems to suggest that, in order of prominence, the hydrophobicity, solvent accessibility, polarity and composition play more prominent role than other feature properties. Hydrophobicity have been shown to be important for metal-protein interactions such that metal binding sites usually appear in clusters with hydrophobic environment. High-affinity metal binding sites in some proteins are located at sequence segments with specific amino acid composition[63], and specific sequence motifs have been used for predicting metal-binding proteins[64,65]. It was also found that polarity and solvent accessibility of the binding site influences the functional properties of metal-binding proteins[66]. Therefore, our prediction results are consistent with these experimental findings.

## Conclusion

SVM appears to be a potentially useful tool for the prediction of metal-binding proteins of different classes. The prediction accuracy may be further enhanced with the further expansion of our knowledge about metal-binding proteins particularly for those small metal-binding classes, more refined representation of the structural and physicochemical properties of proteins, and the improvement of prediction algorithms such as the better treatment of imbalanced dataset. The SVM-derived metal-binding protein classification systems developed in this work can be assessed, free of charge for academic use, at the SVMProt server [67].

## Methods

### Selection of metal-binding and non-metal-binding proteins

All metal-binding proteins used in this study are collected from a comprehensive search of Swiss-Prot database [42]. A total of 33295 metal-binding protein sequences were obtained. Most of these proteins can be classified into one of the 10 metal-binding classes, and the number of proteins is 5426, 1467, 1645, 9462, 9688, 4214, 705, 1240, 1567 and 16072 in calcium-binding, cobalt-binding, copper-binding, iron-binding, magnesium-binding, manganese-binding, nickel-binding, potassium-binding, sodium-binding and zinc-binding class respectively. Some proteins were found to belong to more than one class. The distribution of all these proteins in different kingdoms and in top 10 host species is given in Table 3, and that of the four largest classes of metal-binding proteins is given in Table 4. From these two Tables one finds that these proteins are from diverse range of species and all species appear to be fairly adequately represented.

All distinct members in each class were used to construct a positive dataset for the corresponding SVM prediction system. A negative dataset, representing non-class members, are selected by a well-established procedure [45,68,69] such that all proteins are grouped into domain families [38] and the representative proteins of those families un-related to the specific metal-binding class are used as negative samples. Members in the other metal-binding classes were included in the negative dataset if they are not a member of the class being studied. These datasets are divided into separate training, testing and independent evaluation sets by the following procedure: First, proteins were clustered into groups based on their distance in the structural and physicochemical feature-space by using the hierarchical clustering method. An upper-limit of the largest separation of 20 was used to for each cluster. One representative protein was randomly selected from each group to form a training set that is sufficiently diverse and broadly distributed in the feature space. One or up to 50% of the remaining proteins in each group were randomly selected to form the testing set. The selected proteins from each group were further checked to ensure that they are distinguished from proteins from other groups. The remaining proteins were used as the independent evaluation set, which are also of reasonable level of diversity. Moreover, an analysis of the "similarity" proteins in each cluster shows that the majority of the proteins in a cluster are non-homologous. Thus, the testing and independent evaluation sets are expected to have certain level of usefulness for performing their task of fine-tuning the parameter of a SVM classification system and for evaluating its prediction performance. The statistics of the members and non-members of the datasets of each metal-binding class is given in Table 1.

### Derivation of structural and physicochemical properties from protein sequence

Construction of the feature vector for each protein is based on the formula used in the prediction of RNA-binding proteins [69], protein-protein interaction[70], protein fold recognition [62], and protein functional family prediction [68]. Given the sequence of a protein, its amino acid composition and the properties of every constituent amino acid are computed and then used to generate this vector. The computed amino acid properties include hydrophobicity, normalized Van der Waals volume, polarity, polarizability, charge, surface tension, secondary structure and solvent accessibility [68].

For each of these properties, amino acids are divided into three groups such that those in a particular group are regarded to have approximately the same property. For instance, amino acids can be divided into hydrophobic (CVLIMFW), neutral (GASTPHY), and polar (RKEDQN) groups. Three descriptors, composition (C), transition (T), and distribution (D), are introduced to describe global composition of each of these properties. C is the number of amino acids of a particular property (such as hydrophobicity) divided by the total number of amino acids in a protein sequence. T characterizes the percent frequency with which amino acids of a particular property is followed by amino acids of a different property. D measures the chain length within which the first, 25%, 50%, 75% and 100% of the amino acids of a particular property is located respectively.

**Figure 1:**
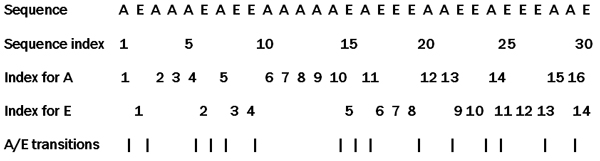
The sequence of a hypothetic protein for illustration of derivation of the feature vector of a protein. Sequence index indicates the position of an amino acid in the sequence. The index for each type of amino acids in the sequence (A or E) indicates the position of the first, second, third, ... of that type of amino acid (The position of the first, second, third, ..., A is at 1, 3, 4, ...). A/E transition indicates the position of AE or EA pairs in the sequence.

A hypothetical protein sequence AEAAAEAEEAAAAAEAEEEAAEEAEEEAAE, as shown in Figure 1, has 16 alanines (n1 = 16) and 14 glutamic acids (n2 = 14). The composition for these two amino acids are n1 × 100.00/(n1 + n2) = 53.33 and n2 × 100.00/(n1 + n2) = 46.67 respectively. There are 15 transitions from A to E or from E to A in this sequence and the percent frequency of these transitions is (15/29) × 100.00 = 51.72. The first, 25%, 50%, 75% and 100% of As are located within the first 1, 5, 12, 20, and 29 residues respectively. The D descriptor for As is thus 1/30 × 100.00 = 3.33, 5/30 × 100.00 = 16.67, 12/30 × 100.00 = 40.0, 20/30 × 100.00 = 66.67, 29/30 × 100.00 = 96.67. Likewise, the D descriptor for Es is 6.67, 26.67, 60.0, 76.67, 100.0. Overall, the amino acid composition descriptors for this sequence are C = (53.33, 46.67), T = (51.72), and D = (3.33, 16.67, 40.0, 66.67, 96.67, 6.67, 26.67, 60.0, 76.67, 100.0) respectively. Descriptors for other properties can be computed by a similar procedure.

Overall, there are 21 elements representing these three descriptors: 3 for C, 3 for T and 15 for D. The feature vector of a protein is constructed by combining the 21 elements of all of these properties and the 20 elements of amino acid composition in sequential order.

There is some level of overlap in the descriptors for hydrophobicity, polarity, and surface tension. Thus the dimensionality of the feature vectors may be reduced by principle component analysis (PCA). Our own study suggests that the use of PCA reduced feature vectors only moderately improves the accuracy for some of the families. It is thus unclear to which extent this overlap affects the accuracy of SVM classification. It is noted that reasonably accurate results have been obtained using these overlapping descriptors in various protein classification studies [62,68,70-72].

### Support Vector Machines method

The algorithms of SVM and its applications to proteins are extensively described in the literature [68,69,73]. Thus only a brief description is given here. A linear SVM constructs a hyperplane that separates two different classes of feature vectors with a maximum margin. One class represents metal-binding proteins and the other non-metal-binding proteins. This hyperplane is constructed by finding a vector **w **and a parameter *b *that minimizes ||**w**||^2 ^which satisfies the following conditions: **w · x**_*i *_+ *b *≥ 1, for *y*_*i *_= +1(positive class) and **w · x**_*i *_+ *b *≤ -1, for *y*_*i *_= -1 (negative class). Here **x**_*i *_is a feature vector, *y_i _*is the group index, **w **is a vector normal to the hyperplane, |*b*|/||**w**|| is the perpendicular distance from the hyperplane to the origin, and ||**w**||^2 ^is the Euclidean norm of **w**.

A nonlinear SVM projects feature vectors into a high dimensional feature space by using a kernel function such as a Gaussian kernel function . The linear SVM procedure is then applied to the feature vectors in this feature space. After the determination of **w **and *b*, a given vector **x **can be classified by using *sign *[(**w · x**) + *b*], a positive or negative value indicates that the vector **x **belongs to the positive or negative class respectively.

The performance of SVM has been measured by the positive, negative and overall prediction accuracies *P_p _= TP/(TP + FN), P_n _= TN/(TN + FP) *and *P = (TP + TN)/N*, which correspond to the accuracies for proteins of a metal-binding class, non-members of the class, and all members and non-members of the class respectively. Here *TP*, *TN, FP*, and *FN *are the number of true positive (true member), true negative (true non-member), false positive (false member), and false negative (false non-member) respectively, and N is the total number of proteins studied.

## Authors' contributions

HH wrote the programs and implemented the study. LY designed the web service. HL, CJ and BX performed the data analysis. ZWC assisted in the study and revised the paper.  YZ conceived of the study and wrote the manuscript. All authors read and approved the final manuscript.

## Figures and Tables

**Table 1:** Statistics of the datasets and prediction accuracy of individual class of metal-binding proteins and that of all metal-binding proteins. The predicted results are given in *TP *(true positive), *FN *(false negative), *TN *(true negative), *FP *(false positive), sensitivity *SE *= *TP*/(*TP *+ *FN*) (accuracy for class members), specificity *SP *= *TN*/(*TN *+ *FP*) (accuracy for non-members), and overall accuracy *Q *= (*TN *+ *TP*)/(*TP *+ *FN *+ *TN *+ *FP*). The number of members and non-members in the testing and independent evaluation sets is *TP *+ *FN *or *TN *+ *FP *respectively

Metal-Binding Protein Classes	Training set		Testing set				Independent evaluation set						
	
	positive	negative	positive		negative		positive			negative			Q(%)
			TP	FN	TN	FP	TP	FN	SE(%)	TN	FP	SP(%)	
Calcium-binding	1816	4389	2055	245	8039	1906	1130	180	86.3%	6373	851	88.2%	87.9%
Cobalt-binding	568	2151	456	2	13407	10	360	81	81.6%	7809	7	99.9%	98.9%
Copper-binding	652	1999	417	109	13115	270	390	77	83.5%	7587	146	98.1%	97.3%
Iron-binding	3128	3428	4869	290	3992	675	1104	71	94.0%	6328	598	91.4%	91.7%
Magnesium-binding	2583	4023	2307	594	7267	115	3412	792	81.2%	5848	805	87.9%	85.3%
Manganese-binding	1608	3099	1146	217	10841	1086	1061	182	85.4%	7148	415	94.5%	93.2%
Nickel-binding	407	2001	95	2	13576	138	156	45	77.6%	7816	65	99.2%	98.6%
Potassium-binding	408	1845	489	10	13789	8	301	32	90.4%	7847	4	99.9%	99.6%
Sodium-binding	777	2010	338	1	13591	30	410	41	90.9%	7831	11	99.9%	99.4%
Zinc-binding	2731	6416	6610	569	5931	360	4616	1546	74.9%	6289	127	98.0%	86.7%
All metal-binding	5013	3101	11806	1015	4217	522	12070	3391	78.1%	4529	617	88.0%	80.6%

**Table 2:** Prediction results of novel metal-binding proteins by SVMProt, where "+" represents proteins correctly predicted as metal-binding proteins, and "-" represents proteins incorrectly predicted as non-metal-binding proteins

**Swiss-Prot AC**	**Prediction Status**	**Swiss-Prot AC**	**Prediction Status**	**Swiss- Prot AC**	**Prediction Status**	**Swiss-Prot AC**	**Prediction Status**
P04390	-	P20910	+	P43589	-	Q09824	-
O13826	-	P22635	-	P49412	+	Q17374	+
O13862	+	P23382	-	P49659	+	Q44009	+
O26638	+	P23485	+	P50534	+	Q45488	+
O29031	+	P23657	-	P52283	-	Q52982	-
O29156	+	P23940	+	P54355	+	Q54450	+
O29747	-	P24005	+	P54657	+	Q56X52	+
O42720	-	P24059	+	P56200	-	Q59660	-
O67672	+	P24282	-	P80479	+	Q6F4C6	+
O68557	+	P26902	+	P80509	-	Q7Z2C4	+
O75448	+	P28875	+	P81040	+	Q80874	+
O81916	+	P31032	+	P81242	-	Q8GNT2	+
P03697	+	P31178	+	P81605	-	Q8VYR2	+
P03825	+	P32505	+	P82604	+	Q94702	+
P12258	+	P33353	+	P83310	-	Q95QY7	-
P12608	-	P33440	+	Q00166	+	Q9JJV3	+
P14229	+	P34806	+	Q00167	-	Q9LAI0	-
P14633	+	P39405	-	Q00457	+	Q9LIG0	+
P19729	+	P40379	+	Q03471	-	Q9VL31	-
P19733	+	P40685	-	Q04580	-	Q9WXE6	+
P20050	-	P40962	+	Q06200	+	Q9ZAA8	+
P20193	-	P40988	+	Q08906	+		

**Table 3:** Distribution of metal-binding proteins in different kingdoms and in top 10 host species of each kingdom. Not all protein sequences studied in this work are included because the host species information of some protein sequences is not yet available in the protein sequence database

**Kingdom**	**Viridae**	**Eukaryota**	**Bacteria**	**Archaea**
**Number of proteins in kingdom**	576	17040	13692	1618
**List of top 10 species and number of proteins in each species**	Bacteriophage T4 (12)	Homo sapiens (2218)	Escherichia coli (551)	Methanococcus jannaschii (203)
	Orgyia pseudotsugata multicapsid polyhedrosis virus (10)	Mus musculus (1850)	Escherichia coli O157:H7 (264)	Methanobacterium thermoautotrophicum(103)
	Autographa californica nuclear polyhedrosis virus (9)	Rattus norvegicus (1013)	Bacillus subtilis (250)	Methanosarcina acetivorans (93)
	Mimivirus (9)	Arabidopsis thaliana (882)	Salmonella typhimurium (229)	Archaeoglobus fulgidus (92)
	Variola virus (6)	Saccharomyces cerevisiae (528)	Escherichia coli O6 (212)	Methanosarcina mazei (91)
	Vaccinia virus (strain Copenhagen) (6)	Drosophila melanogaster (455)	Haemophilus influenzae (205)	Halobacterium salinarium (75)
	Vaccinia virus (strain Western Reserve/WR) (6)	Caenorhabditis elegans (388)	Shigella flexneri (197)	Pyrococcus horikoshii (72)
	Vaccinia virus (strain Ankara) (6)	Bos Taurus (334)	Salmonella typhi (173)	Pyrococcus abyssi (71)
	Ictalurid herpesvirus 1 (5)	Schizosaccharom yces pombe (314)	Mycobacterium tuberculosis (164) Synechocystis sp.	Pyrococcus furiosus (70)
	African swine fever virus (strain BA71V) (5)	Gallus gallus (252)	(strain PCC 6803) (163)	Sulfolobus solfataricus (65)

**Table 4:** Distribution of different classes of metal-binding proteins (calcium-binding, magnesium-binding, iron-binding, and zinc-binding) in different kingdoms and in top 10 host species. Not all protein sequences studied in this work are included because the host species information of some protein sequences is not yet available in the protein sequence database

	Calcium-binding		Magnesium-binding		Iron-binding		Zinc-binding	
	
	Kingdom or species	No. of proteins	Kingdom or species	No. of proteins	Kingdom or species	No. of proteins	Kingdom or species	No. of proteins
**Protein distribution in kingdom**	Archaea	73	Archaea	262	Archaea	381	Archaea	1048
	Bacteria	1092	Bacteria	2597	Bacteria	3743	Bacteria	6916
	Eukaryota	3897	Eukaryota	1081	Eukaryota	5248	Eukaryota	6464
	Viridae	343	Viridae	194	Viridae	29	Viridae	1466
**Protein distribution in top 10 species**	Homo sapiens	651	Homo sapiens	140	Arabidopsis thaliana	278	Homo sapiens	1121
	Mus musculus	499	Mus musculus	129	Escherichia coli	214	Mus musculus	911
	Rattus norvegicus	305	Arabidopsis thaliana	117	Homo sapiens	191	Rattus norvegicus	382
	Arabidopsis thaliana	186	Rattus norvegicus	69	Mus musculus	185	Saccharomyces cerevisiae	380
	Bos taurus	142	Escherichia coli	63	Rattus norvegicus	152	Arabidopsis thaliana	359
	Gallus gallus	103	Saccharomyces cerevisiae	55	Drosophila melanogaster	124	Caenorhabditis elegans	255
	Drosophila melanogaster	94	Bacillus subtilis	53	Methanococcus jannaschii	92	Drosophila melanogaster	237
	Oryctolagus cuniculus	82	Escherichia coli O157:H7	45	Saccharomyces cerevisiae	88	Schizosaccharomyces pombe	221
	Sus scrofa	65	Salmonella typhimurium	44	Escherichia coli O157:H7	87	Escherichia coli	172
	Caenorhabditis elegans	64	Schizosaccharomyces pombe	43	Bacillus subtilis	77	Methanococcus jannaschii	119
